# Advancing the science and practice of primary health care as a foundation for universal health coverage: a call for papers

**DOI:** 10.2471/BLT.19.239889

**Published:** 2019-08-01

**Authors:** Etienne V Langlois, Shannon Barkley, Edward Kelley, Abdul Ghaffar

**Affiliations:** a Alliance for Health Policy and Systems Research, Science Division, World Health Organization, avenue Appia 20, 1211 Geneva 27, Switzerland.; b Integrated Health Services, Universal Health Coverage and Life Course, World Health Organization, Geneva, Switzerland.

Health systems face increasingly complex challenges, such as the growing burden of chronic noncommunicable diseases, multisource pollution, new epidemics and antimicrobial resistance. These challenges have prompted an important shift in focus from curative care to health promotion and disease prevention, as well as the development of new models of service delivery, financing and governance for primary health care.

Global health stakeholders are pushing for renewed commitments to enhance primary health care in the 21st century, in light of the 2018 Declaration of Astana.^1^ The 2019 World Health Assembly adopted an ambitious resolution recognizing the role of primary health care in providing the full range of health services needed throughout the life course, including prevention, treatment, rehabilitation and palliative care.^2^

Achieving the health-related sustainable development goals (SDGs), including universal health coverage (UHC), will not be possible without stronger primary health care.^3^ Action is required to strengthen the three pillars of primary health care, that is, primary care and essential public health functions as the core of integrated health services; empowered people and communities; and multisectoral policy and action.^1^

Most people not covered by essential health services belong to disadvantaged groups. Primary health care can address this issue because it enhances equity and is the most feasible approach to reach people.^4^^–^^6^ Strengthening primary health care is a cost–effective strategy for UHC^7^ and is a strong entry point to improving high-quality health systems.^8^ Primary health-care systems are also well positioned to address evolving population needs and contribute to the responsiveness and resilience of health systems.^8^

However, in many low- and middle-income countries, primary health-care systems are weak and do not provide sufficient high-quality, comprehensive, people-centred and integrated care. In addition, the core principles of empowering people and strengthening health systems are often overshadowed by short-term interventions, with countries attempting to meet an overwhelming number of programme-specific targets that are dependent on donor priorities and funding. This situation highlights the need to strengthen comprehensive primary health-care systems based on local priorities, needs and contexts. These systems should be co-developed by people who are engaged in their own health.

Implementation and systems reforms of primary health care are also challenged by the lack of contextualized knowledge and scarcity of research on effective and acceptable approaches to strengthen primary health care, especially in low- and middle-income countries. Primary health-care research that is applicable to different health systems settings is needed.

To tackle these challenges, the *Bulletin of the World Health Organization* will publish a theme issue on advancing the science and practice of primary health care as a foundation for UHC. The theme issue aims at providing a forward-looking view of the innovations, challenges and shared responsibilities in driving primary health care, and at filling gaps and fostering discussions around what is needed to implement a primary health-care vision towards UHC. 

We welcome manuscripts that capture learnings and experience in the implementation of primary health-care policies and interventions as well as the strengthening of primary health-care systems. The ability of countries to fulfil the commitments made in the Declaration of Astana depends on the active engagement of various stakeholders of primary health care. We therefore encourage analysis of challenges, contextualized research, good practices and innovations in enhancing primary health care at the national or sub-national level, with a strong focus on stakeholder engagement and impact on population health and equity. Papers could focus on, for example, multisectoral action for primary health care and equity, implementation research and analysis of health systems reforms, bridging the gaps in workforce, digital health, quality improvement and the contribution of primary health care in advancing UHC and the SDGs.

Documenting good practices on effective approaches to engage and empower people, communities, youth and other stakeholders will also be useful to support efforts enhancing the responsiveness and people-centeredness of primary health care. The theme issue will focus on cross-cutting themes and challenges of primary health care affecting all countries, but we also encourage submission of experiences from low- and middle-income countries and conflict-affected and humanitarian settings, as well as comparative cross-country analyses.

The deadline for submission is 30 November 2019. Manuscripts should be submitted in accordance with the *Bulletin*’s guidelines for contributors (available at: http://www.who.int/bulletin/volumes/96/1/18-990118/en/) and the cover letter should mention this call for papers.

This theme issue will be launched at the Global Symposium on Health Systems Research in Dubai in 2020.
